# Trustworthy navigation with variational policy in deep reinforcement learning

**DOI:** 10.3389/frobt.2025.1652050

**Published:** 2025-10-08

**Authors:** Karla Bockrath, Liam Ernst, Rohaan Nadeem, Bryan Pedraza, Dimah Dera

**Affiliations:** 1 Chester F. Carlson Center for Imaging Science, Rochester Institute of Technology, Rochester, NY, United States; 2 Department of Electrical and Computer Engineering, The University of Texas Rio Grande Valley, Edinburg, TX, United States

**Keywords:** deep reinforcement learning, robot uncertainty, trustworthy navigation, variational policy, moment propagation

## Abstract

**Introduction:**

Developing a reliable and trustworthy navigation policy in deep reinforcement learning (DRL) for mobile robots is extremely challenging, particularly in real-world, highly dynamic environments. Particularly, exploring and navigating unknown environments without prior knowledge, while avoiding obstacles and collisions, is very cumbersome for mobile robots.

**Methods:**

This study introduces a novel trustworthy navigation framework that utilizes variational policy learning to quantify uncertainty in the estimation of the robot’s action, localization, and map representation. Trust-Nav employs the Bayesian variational approximation of the posterior distribution over the policy-based neural network’s parameters. Policy-based and value-based learning are combined to guide the robot’s actions in unknown environments. We derive the propagation of variational moments through all layers of the policy network and employ a first-order approximation for the nonlinear activation functions. The uncertainty in robot action is measured by the propagated variational covariance in the DRL policy network. At the same time, the uncertainty in the robot’s localization and mapping is embedded in the reward function and stems from the traditional Theory of Optimal Experimental Design. The total loss function optimizes the parameters of the policy and value networks to maximize the robot’s cumulative reward in an unknown environment.

**Results:**

Experiments conducted using the Gazebo robotics simulator demonstrate the superior performance of the proposed Trust-Nav model in achieving robust autonomous navigation and mapping.

**Discussion:**

Trust-Nav consistently outperforms deterministic DRL approaches, particularly in complicated environments involving noisy conditions and adversarial attacks. This integration of uncertainty into the policy network promotes safer and more reliable navigation, especially in complex or unpredictable environments. Trust-Nav offers a step toward deployable, self-aware robotic systems capable of recognizing and responding to their own limitations.

## 1 Introduction

Autonomous mobile robots are designed to execute complex tasks, navigate, and interact with unknown real-world environments. However, the challenges posed by the dynamic nature of the real world introduce a spectrum of obstacles that require innovative solutions (Wong et al., 2018; Carter-Templeton et al., 2018; Liaqat et al., 2019; Alatise and Hancke, 2020; Nam and Gon-Woo, 2021; Niloy et al., 2021; Gupta and Fernando, 2022; Wijayathunga et al., 2023). From surviving unpredictable barriers to responding to noisy or attacked environmental conditions, these challenges underscore the complexity of achieving autonomy in mobile robotic systems.

Deep reinforcement learning (DRL), rooted in the synergy of deep neural networks (DNNs) and reinforcement learning (RL) principles, has emerged as a powerful paradigm to endow autonomous robotic systems with adaptive and intelligent navigation and decision-making capabilities (Mnih et al., 2015; Wang et al., 2016; Gu et al., 2017; Zambaldi et al., 2018; Liu R. et al., 2021; Plaat, 2022). DRL offers a promising avenue for imbuing robots with the capability to learn and optimize behaviors autonomously with considerable success across various research domains, including navigation and mapping, as a particularly noteworthy area of exploration (Ahmed et al., 2023; Placed et al., 2023). Autonomous navigation encompasses a suite of methodologies wherein a mobile robot not only localizes itself but also concurrently traverses and maps an unfamiliar environment. This dynamic field within RL demonstrates the potential for robotic systems to autonomously navigate and explore unknown spaces while simultaneously building a coherent map of their surroundings. The latter process is known as active simultaneous localization and mapping (SLAM) (Leung et al., 2008; Trivun et al., 2015; Palomeras et al., 2019; Chen et al., 2020; Mihálik et al., 2022; Ahmed et al., 2023; Placed et al., 2023).

This paper proposes a novel trustworthy navigation (Trust-Nav) framework that adopts DRL and develops a variational policy learning paradigm. The variational policy consists of a Bayesian policy neural network, where we define a prior distribution over the parameters of the policy network. When the robot receives observations from the environment, the distribution over the parameters is updated to the posterior distribution using Bayes’ rule. However, computing the exact posterior is often intractable due to the complexity and high dimensionality of neural networks. We approximate the posterior distribution of the policy network’s parameters using variational inference (Blei et al., 2017). The variational inference framework addresses this difficulty by approximating posterior estimation as an optimization problem, where a simpler distribution (i.e., Gaussian) is optimized to closely match the true posterior. To complete the Bayesian network structure, we propagate the moments of the Gaussian variational posterior through the network layers and estimate the mean and covariance of the predicted robot’s actions of the policy network. The propagated covariance represents the uncertainty associated with the action and is used in the loss function to inform the decision. Moreover, Trust-Nav also computes uncertainty in the robot’s localization and mapping using the D-optimal method (Rodríguez-Arévalo et al., 2018; Placed and Castellanos, 2022) that captures the global variance of the map by analyzing the total length of the covariance of the state vectors. The proposed framework can be applied to various DRL algorithms and produces improved robustness in autonomous robot navigation, especially in noisy environments. The main contributions can be summarized as follows.

• Develop a novel DRL-based trustworthy, reliable, and collision-free autonomous navigation (Trust-Nav) framework that introduces closed-form variational moment propagation into DRL policy networks, and integrates statistical uncertainty in Bayesian theory to guide the robot’s actions and mappings for trustworthy navigation.

• Eliminate MC sampling to overcome robustness and scalability limitations of existing Bayesian DRL approaches, providing a tractable, analytically grounded framework that balances theoretical soundness with the computational constraints of embedded robotic systems.

• Combine policy-based and value-based learning and quantify the uncertainty in the robot’s actions and localizations to guide the navigation toward maximizing cumulative reward.

• Design a Bayesian policy neural network that propagates the mean and covariance of the variational posterior distribution and produces robot actions to the environment and associated uncertainty within each action to guide the robot’s decision-making process.

• Adopt a reward function that accounts for the robot’s localization uncertainty. Both action and localization/mapping uncertainties are combined into a unified loss function to maximize the cumulative reward.

• Assess the Trust-Nav model performance and robustness under various noisy and attacked environments by an adversary using the Gazebo robotics simulator.

## 2 Literature review

### 2.1 Deep reinforcement learning for navigation

Deep Reinforcement Learning (DRL) enables an autonomous robot to learn optimal behaviors through trial-and-error interactions with its environment. In the context of navigation and exploration, the robot—equipped with sensors such as LiDAR and/or cameras—learns to perceive, explore, and map previously unknown environments by leveraging action–feedback loops to iteratively refine its policy (Mnih et al., 2015; Morales et al., 2021; Plaat, 2022).

The robot refines its behavior by receiving rewards or penalties based on the outcomes of its actions, as specified by a developer-defined reward function. Although the reward signal provides some supervision, as it guides the robot toward optimal actions, the robot primarily learns through its own interactions with the environment, making DRL a form of semi-supervised learning. This framework is particularly effective in complex environments characterized by high-dimensional state and action spaces. For example, in tasks such as playing chess, the robot must reason over an enormous decision space to win. To manage such complexity, DRL integrates deep neural networks, which enable the robot to approximate complex, non-linear functions and make decisions in high-dimensional environments. This learning paradigm closely resembles human learning through trial and error, as observed in the process of mastering strategic games such as checkers or chess.

A variety of DRL architectures have been applied to robotics navigation, including value-based methods such as Q-learning (Jang et al., 2019) and Deep Q-Networks (DQN) (Mnih et al., 2015), as well as their enhancements—double DQN (Van Hasselt et al., 2016) and dueling architectures (Wang et al., 2016). While these approaches perform well in discrete action spaces, robotics often requires continuous control of motion parameters such as linear and angular velocities. Policy gradient methods, particularly the Advantage Actor–Critic (A2C) framework (Grondman et al., 2012; Mnih et al., 2016; Grigsby et al., 2021), address this by decoupling policy learning (actor) from value estimation (critic), enabling better action prediction in continuous or mixed action spaces.

Despite these advances, current DRL navigation frameworks remain limited in their ability to operate reliably in real-world conditions where sensor noise, environmental uncertainty, and adversarial disturbances are prevalent. Recent work in robust reinforcement learning has explored adversarial training (Pinto et al., 2017), distributional RL (Liu Q. et al., 2021; Bellemare et al., 2023), and domain randomization (Tobin et al., 2017) to improve robustness, while adaptive control theory (Zhou, 1998) provides decades of insight into stability under uncertainty. However, these strategies often lack explicit mechanisms for quantifying and propagating uncertainty in the decision-making process.

Bayesian neural networks (BNNs) offer a principled approach to uncertainty quantification by modeling distributions over network parameters (Gal and Ghahramani, 2016; Kendall and Gal, 2017; Feng et al., 2019). In robotics, BNNs have been applied to perception (Dera et al., 2021) and control (Wang et al., 2024), demonstrating improved robustness to noisy inputs. Yet, integrating BNNs directly into DRL navigation pipelines remains underexplored. Most uncertainty-aware navigation methods either rely on sampling-based approximations or heuristic measures of prediction confidence, which can be computationally costly or unreliable in safety-critical scenarios.

Our proposed Trust-Nav framework addresses this gap by analytically propagating both the mean and covariance of the variational posterior through the policy network, enabling real-time, self-assessed navigation without additional sampling or computation. This design allows the robot to detect low-confidence decision states and adapt its behavior accordingly, bridging Bayesian uncertainty modeling with DRL and drawing conceptual parallels to robust and adaptive control principles.

### 2.2 Reward computation for navigation

An important component of autonomous exploration is the computation of rewards that guide the robot from its current position toward informative future locations. Prior work has shown that reward design can be grounded in the uncertainty of the robot’s pose and the environment map, encouraging actions that reduce this uncertainty (Carrillo et al., 2012; Rodríguez-Arévalo et al., 2018). Many of these methods are rooted in the Theory of Optimal Experimental Design (TOED) (Pukelsheim, 2006), which provides optimality criteria for selecting actions that maximize the information gained from new observations.

The research community has explored several TOED criteria, including T-optimality, A-optimality, D-optimality, E-optimality, and Shannon’s entropy (Carrillo et al., 2012; Rodríguez-Arévalo et al., 2018; Placed and Castellanos, 2022; Placed and Castellanos, 2020), each emphasizing different statistical properties of the state covariance matrix to infer the uncertainty in the robot’s localization and mapping. For example, A-optimality minimizes the trace of the covariance (average variance), while E-optimality minimizes the maximum eigenvalue (worst-case variance). D-optimality, in contrast, maximizes the determinant of the information matrix (or equivalently, minimizes the volume of the confidence ellipsoid), thereby capturing global variance reduction across all state dimensions. This property makes D-optimality well-suited for active SLAM and exploration, where the objective is to efficiently reduce uncertainty throughout the map rather than along a single dimension.

In this paper, we adopt the D-optimal method as the most effective because its ability to integrate information from all map landmarks (the global variance of the map), represented by the eigenvalues $\lambda_{i}$ of the state covariance matrix $\Sigma_{s}\in R^{d\times d}$ , where $s={\left(s_{1},\ldots ,s_{d}\right)}^{T}$ denotes the state vector. The D-optimal criterion $f_{D}$ is defined in (Equation 1).

$$f_{D}\left(\Sigma_{s}\right)≜\exp\left(\frac{1}{d}\overset{d}{\underset{i=1}{\sum }}\log\left(\lambda_{i}\right)\right).$$ (1)

The D-optimal function has been shown in prior robotics literature (Carrillo et al., 2012; Rodríguez-Arévalo et al., 2018; Placed and Castellanos, 2022; Placed and Castellanos, 2020) to yield more balanced exploration trajectories compared to alternative criteria. The logarithmic formulation prevents convergence to zero, ensuring numerical stability while providing a robust measure of global uncertainty for navigation, exploration, and mapping. The robot communicates back and forth with the environment to help create the map and positions using measurements from LiDAR or camera through the ROS framework (Macenski et al., 2022).

## 3 Trust-navigation with variational policy

The proposed Trust-Nav adopts the policy-value DRL algorithm with deep neural networks to define the robot. The robot was equipped with a depth camera and LiDAR. The camera’s depth images or frames serve as inputs to the DRL neural networks, which determine the best action based on the environment’s state. While LiDAR could be used for input, the camera was found to be more suitable for object detection and avoidance along the robot’s trajectory path.

To extract useful information from images or frames, we deploy two convolutional neural networks (CNNs) for the policy and value functions, respectively. The policy CNN takes the environment states as input and produces probabilistic actions. At the same time, the value function determines the expected return for a robot starting at a given state and acting according to a particular policy. The two networks interact with each other through the temporal difference (TD) learning method, where the policy network makes an action, and the value network returns a value to penalize incorrect actions.

### 3.1 Variational policy network

We develop the policy as a Bayesian CNN with L layers, and the probabilistic network parameters are $W={\left\{W^{\left(l\right)}\right\}}_{l=1}^{L}$ , where $W^{\left(l\right)}$ is the weight matrix for the $l^{\text{th}}$ layer. The Bayesian CNN architecture follows (Dera et al., 2021). We introduce a prior Gaussian distribution over the network parameters, $W∼N_{p}\left(0,cI\right)$ , where c is a hyperparameter that refers to the prior variance. The input-output dataset for the policy network consists of states from the environment and the robot’s actions at time t , i.e., $D_{t}={\left\{s_{t},a_{t}\right\}}_{t=1}^{T}$ . Given the data and the prior, we approximate the posterior distribution of the parameters given the data, i.e., $p\left(W|D_{t}\right)$ by the variational Gaussian distribution $W∼q_{\phi }\left(W\right)=N_{v}\left(\mu ,\Sigma \right)$ . The variational parameters ϕ={μ,Σ} with the mean, μ , and covariance, Σ , are optimized by minimizing Kullback-Leibler (KL) divergence between the approximate and the true unknown posterior $\text{KL}\left[N_{v}\left(\mu ,\Sigma \right)\|p\left(W|D_{t}\right)\right]$ or equivalently maximizing the evidence lower bound (ELBO) loss function that converges to the optimal variational density (Blei et al., 2017). The ELBO loss is defined in (Equation 2).

$$L\left(\mu ,\Sigma ;a_{t}|s_{t}\right)=E_{N_{v}}\left(\log p\left(a_{t}|s_{t},W\right)\right)-\text{KL}\left[N_{v}\|N_{p}\right].$$ (2)

The ELBO loss function consists of two terms: (i) the expected log-likelihood of the robot’s actions given the environment states and the probabilistic weights, and (ii) the regularization term, which is the KL divergence between the variational posterior and prior Gaussian distributions. The likelihood of the actions given the states, $p\left(a_{t}|s_{t},W\right)$ , is modeled by a Gaussian distribution with the action’s mean, $\mu_{a_{t}}$ , and covariance, $\Sigma_{a_{t}}$ , predicted at the output of the variational policy network. We approximate the expectation over the variational posterior in the first term of the ELBO loss using the first-order Taylor approximation as defined in (Equation 3). The use of a first-order Taylor expansion is a deliberate choice to enable closed-form propagation of both the mean and covariance of the variational posterior through nonlinear activation functions. This choice allows us to model the full predictive distribution in an analytically tractable manner, entirely avoiding the need for Monte Carlo (MC) sampling. MC-based uncertainty estimation, while accurate in theory, is computationally expensive, introduces sampling noise, and scales poorly with deeper network architectures—limitations that are particularly critical in real-time robotics. The first-order approach therefore strikes a balance between accuracy and scalability, making it feasible to propagate uncertainty through deeper policy networks and to support low-latency inference. Although the first-order approximations can accumulate error in deep networks, our empirical results demonstrate that even with this assumption, the proposed framework significantly improves both accuracy and robustness compared to deterministic baselines and sampling-based Bayesian DRL approaches. This finding supports the suitability of the first-order approach in practical, real-time navigation scenarios.

We assume that the probabilistic parameters of the policy network are independent within and across layers. This independence assumption is crucial for developing a feasible optimization problem in high-dimensional policy networks. Estimating and storing a full covariance matrix across all weights is not computationally or mathematically tractable for large-scale DRL models, where the parameter count can be in the millions. Furthermore, this independence assumption promotes the extraction of non-correlated, informative features and reduces redundancy, which is beneficial for both generalization and interpretability (Yang et al., 2008). Thus, the variational covariance of the weight vector $w^{\left(l\right)}$ in the l th layer can be written as $\Sigma^{\left(l\right)}=\sigma^{\left(l\right)}I$ , where I is an identity matrix and $\sigma^{\left(l\right)}$ the learnable variance. The second term of the ELBO loss has a closed-form mathematical formulation and can be written as in Equation 4, where $H_{l}$ and $H_{l-1}$ represent the number of neurons in the l th and (l−1) th layers, respectively.

$$E_{W∼N_{v}}\left(\log p\left(a_{T}|s_{T},W\right)\right)\approx -\frac{1}{2T}\overset{T}{\underset{t=1}{\sum }}\left[\log\left(\left|\Sigma_{a_{t}}\right|\right)+{\left(a_{t}-\mu_{a_{t}}\right)}^{T}{\left(\Sigma_{a_{t}}\right)}^{-1}\left(a_{t}-\mu_{a_{t}}\right)\right].$$ (3)

$$\text{KL}\left[N_{v}\|N_{p}\right]=\frac{1}{2}\overset{L}{\underset{l=1}{\sum }}\overset{H_{l}}{\underset{i=1}{\sum }}\left(\|\mu_{i}^{\left(l\right)}{\|}_{F}^{2}-H_{l-1}\left(1-\frac{\sigma_{i}^{\left(l\right)}}{c}+\log\frac{\sigma_{i}^{\left(l\right)}}{c}\right)\right).$$ (4)

Thus, $\mu_{a_{t}}$ and $\Sigma_{a_{t}}$ represent the probabilistic action mean vector and covariance matrix. While $\mu_{i}^{\left(l\right)}$ and $\sigma_{i}^{\left(l\right)}$ are the mean vector and covariance matrix of the variational posterior distribution over the policy neural network’s parameters for the i th weight vector, $w_{i}^{\left(l\right)}$ , in the l th layer.

### 3.2 Policy variational moments propagation

In the proposed framework, the parameters of the policy network are modeled as probabilistic variables, specifically following Gaussian distributions. To accommodate this formulation, all network layers are redefined such that their computations operate on these probabilistic parameters. The variance values associated with the Gaussian posterior distributions capture the uncertainty in the model parameters. This parameter uncertainty propagates through the network layers, ultimately enabling the estimation of uncertainty in the robot’s actions at the output of the policy network. Although the network parameters are assumed to be independent across layers, the output of each layer exhibits non-trivial correlations due to the transformations applied during forward propagation. Thus, the covariance over the output of every layer exists through the mathematical derivation. To quantify the uncertainty at each stage of the network—including convolutional layers, multi-layer perceptrons, and non-linear activation functions—we derive the output distributions using statistical properties of random variable transformations and the first-order (e.g., Taylor series) approximation.

The convolution and fully connected layers can be expressed as a multiplication between the input matrix X and the weight matrix W , i.e., Z=XW . The input matrix $X\in R^{n\times d}$ has n probabilistic feature vectors (random vectors) as rows $x_{i}^{T}\in R^{1\times d}$ , and $W\in R^{d\times m}$ has m probabilistic weight vectors as columns $w_{j}\in R^{d}$ . The mean matrix of the input feature vectors, where the means of the feature vectors are arranged in the matrix’s rows, is given in Equation 5.

$$M^{\left(x\right)}=\left[\begin{array}{c} \mu_{x_{1}}^{T}\\ \mu_{x_{2}}^{T}\\ \vdots \\ \mu_{x_{n}}^{T} \end{array}\right]\in R^{n\times d}$$ (5)

where $\mu_{x_{i}}=E\left[x_{i}\right]$ is the mean of the i th row vector. The covariance matrix associated with each $x_{i}$ , denoted by $\Sigma_{x_{i}}\in R^{d\times d}$ is defined as $\Sigma_{x_{i}}=E\left[\left(x_{i}-\mu_{x_{i}}\right){\left(x_{i}-\mu_{x_{i}}\right)}^{T}\right]$ .

Similarly, every column of the matrix W is $w_{j}$ , and the mean matrix of the weights, which is the matrix of the mean vectors arranged in its columns, is defined as $M^{\left(w\right)}=\left[\mu_{w_{1}}\;\mu_{w_{2}}\;\cdots \;\mu_{w_{m}}\right]\in R^{d\times m}$ with $\mu_{w_{j}}=E\left[w_{j}\right]$ and the covariance matrix $\Sigma_{w_{j}}\in R^{d\times d}$ , where $i,\;j=1,\ldots ,H_{l}$ .

To simplify notation for the covariance derivation, we vectorize the output matrix Z into a single column vector: $z=vec\;\left(Z\right)=vec\;\left(XW\right)\in R^{mn\times 1}$ , where vec is the vectorization operation and the (i,j) -th element of Z (row i , column j ) appears in z at position (i−1)m+j .

**FIGURE 1:**
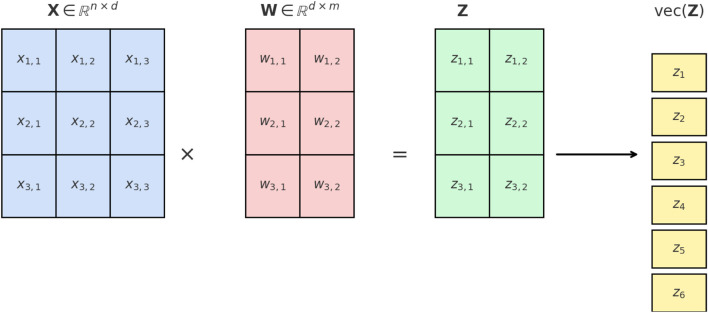
Illustration of the vectorization process in the covariance propagation derivation. The input matrix $X\in R^{n\times d}$ multiplies the weight matrix $W\in R^{d\times m}$ to yield $Z=XW\in R^{n\times m}$ , where each entry is $z_{i,j}=x_{i}^{⊤}w_{j}$ . The vectorization $z=vec\left(Z\right)\in R^{nm\times 1}$ stacks row vectors of Z , so $z_{i,j}$ maps to position (i−1)m+j in z . This explicit index mapping preserves the correspondence between each scalar and its originating pair $\left(x_{i},w_{j}\right)$ , enabling consistent computation of means and covariances.

This ordering ensures that the indices i (input row) and j (weight column vector) are explicitly preserved, so that the mean and covariance for each element of z can be expressed in terms of the corresponding $x_{i}$ and $w_{j}$ . Thus, the mean and covariance of the output vector z are derived in Equation 6 following the multiplication between two random vectors. Tr represents the matrix trace.

$$\begin{array}{cc} \mu_{z} & =vec\left(M^{\left(x\right)}M^{\left(w\right)}\right),\\ \Sigma_{z}\left[p,q\right] & =\left\{\begin{array}{cc} \text{Var}\left(x_{i}^{T}\;w_{j}\right),\; & \text{if }p=q\\ \text{Cov}\left(x_{i}^{T}\;w_{j},x_{i^{\prime }}^{T}\;w_{j^{\prime }}\right),\; & \text{if }p\neq q \end{array}\right. \end{array}$$ (6)

where the index mapping p↔(i,j) and $q↔\left(i^{\prime },j^{\prime }\right)$ follows the vectorization ordering above. Under the independence assumption between $x_{i}$ and $w_{j}$ the variance of each element is simplified in Equation 7.

$$\Sigma_{z}\left[p,q\right]=\left\{\begin{array}{cc} \text{Var}\left(x_{i}^{T}\;w_{j}\right)=Tr\left(\Sigma_{x_{i}}\Sigma_{w_{j}}\right)+\mu_{x_{i}}^{⊤}\Sigma_{w_{j}}\mu_{x_{i}}+\mu_{w_{j}}^{⊤}\Sigma_{x_{i}}\mu_{w_{j}},\; & \text{if }p=q\\ \text{Cov}\left(x_{i}^{T}\;w_{j},x_{i^{\prime }}^{T}\;w_{j^{\prime }}\right)=Tr\left(\Sigma_{x_{i}}\Sigma_{w_{j}}\right)+\mu_{x_{i}}^{⊤}\Sigma_{w_{j}}\mu_{x_{i^{\prime }}}+\mu_{w_{j}}^{⊤}\Sigma_{x_{i}}\mu_{w_{j^{\prime }}},\; & \text{if }p\neq q \end{array}\right.$$ (7)

where the indices $i=i^{\prime }$ and $j=j^{\prime }$ provide the variance components of the matrix $\Sigma_{z}$ and the indices $i\neq i^{\prime }$ and $j\neq j^{\prime }$ provide the covariance components. Figure 1 illustrates the vectorization process in the covariance propagation derivation.

The mean and covariance at the output of the activation function, y=F(z) , are derived using the first-order Taylor approximation as in Equation 8.

$$\mu_{y}\approx F\left(\mu_{z}\right);\;\Sigma_{y}\approx J_{F}\;\Sigma_{z}\;J_{F}^{T},$$ (8)

where $J_{F}$ represents the Jacobian matrix of the activation function F with respect to the input vector z , evaluated at the mean $\mu_{z}$ .

### 3.3 Value network and reward design

We define the value function as a CNN that takes the state and reward from the environment as inputs and produces the value estimate that penalizes the robot’s incorrect actions. The parameters of the value network are $U={\left\{U^{\left(l_{v}\right)}\right\}}_{l_{v}=1}^{L_{v}}$ , where $U^{\left(l_{v}\right)}$ are the weight matrices for $l_{v}=1,\ldots ,L_{v}$ layers. The critic or penalty value estimates, $V\left(s_{t},U\right)$ , serve as a baseline for the policy network to update its parameters through policy-gradient approach and back-propagation (Sewak, 2019). The temporal difference (TD) error, $\delta_{t}$ , between the subsequent state-value estimates is computed using the instantaneous reward and discounted state value of the subsequent state as in (Equation 9), where γ is the discounting factor and $r_{t}\left(s_{t},a_{t}\right)$ is the reward. $\delta_{t}$ in (Equation 9) represents one-step return updates, which can be expanded to a multi-step update. The value function in (Equation 9), $V\left(s_{t};U\right)$ , is the state-value CNN estimator parametrized by weights or parameters U .

$$\delta_{t}=r_{t}\left(s_{t},a_{t}\right)+\gamma V\left(s_{t+1};U\right)-V\left(s_{t};U\right).$$ (9)

**FIGURE 2:**
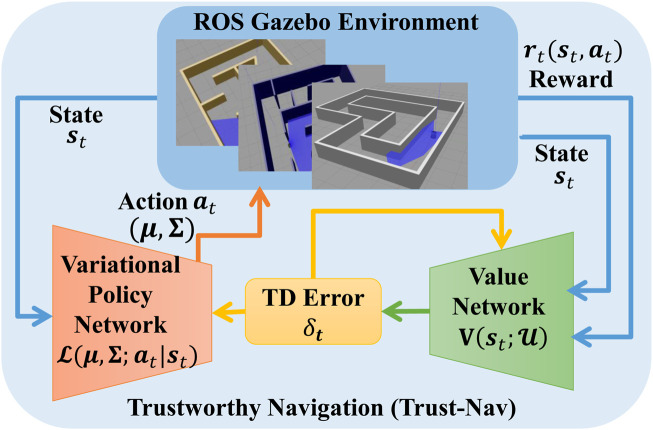
The proposed Trust-Nav framework with the variational policy and value networks forming a robot that interacts with the environment, and quantifies uncertainty in the robot’s actions.

Figure 2 illustrates the general structure of the proposed Trust-Nav framework, where the policy and value networks form a robot that interacts with the environment. The detailed interaction and optimization procedure is provided in Algorithm 1.

### 3.4 Reward function

The reward function defined in Equation 10 incorporates a standard non-collision mechanism that imposes strong penalties on the robot for collisions or other undesirable behaviors with an exploration bonus grounded in D-optimality from TOED. Collisions or unsafe actions incur a large negative reward, while forward motion without collision receives the highest positive reward, turning receives a smaller positive reward, and exploration into unmapped areas receives an additional uncertainty-based reward.

To encourage informative exploration, we employ the D-optimality criterion from the Theory of Optimal Experimental Design (TOED). In TOED, D-optimality maximizes the determinant of the information matrix, which is equivalent to minimizing the volume of the pose-map confidence ellipsoid associated with the estimated parameters. In the context of active SLAM and exploration, this property directly translates to maximizing global information gain about the environment and reducing overall localization and mapping uncertainty. Compared to other TOED measures such as A-optimality (which minimizes the average variance) or E-optimality (which minimizes the maximum or worst-case variance), D-optimality captures global variance across all state dimensions and has been shown in prior work (Carrillo et al., 2012; Rodríguez-Arévalo et al., 2018) to produce more balanced and efficient exploration trajectories in robotics.

The exploration reward is bounded using the hyperbolic tangent function, tanh(.) , with scaling factor ζ , to prevent extreme exploration values from dominating the fixed forward/turn rewards. This normalization strategy stabilizes learning and is consistent with reward-bounding methods used in reinforcement learning for navigation. This structured reward design encourages safe navigation while explicitly rewarding globally informative exploration, thus promoting efficient and robust policy learning. The reward function is defined in Equation 10.

$$r_{t}\left(s_{t},a_{t}\right)=\left\{\begin{array}{cc} -100,\; & \text{if collision}\\ 1+\tanh\left(\frac{\zeta }{f_{D}\left(\Sigma_{s_{t}}\right)}\right),\; & \text{if straight}\\ -0.1+\tanh\left(\frac{\zeta }{f_{D}\left(\Sigma_{s_{t}}\right)}\right)\; & \text{if turning}, \end{array}\right.$$ (10)

where ζ is a task-dependent scale factor and $f_{D}\left(\Sigma_{s_{t}}\right)$ is the D-optimality criterion. The D-optimal exploration reward is derived from TOED (Placed and Castellanos, 2022). This design of the reward components follows standard practices in reinforcement learning for navigation tasks, where the goal is to balance safety, efficiency, and exploration.

• Collision penalty (-100): A large negative reward is assigned to collisions to strongly discourage unsafe behaviors. This magnitude is consistent with navigation benchmarks, where collisions must be treated as catastrophic outcomes relative to other objectives.

• Straight motion reward (+1): A positive baseline reward is assigned to forward movement to encourage progress toward the goal and avoid oscillatory or stagnant behaviors.

• Turning penalty (-0.1): A small negative reward is assigned to turning to discourage excessive rotations without progress. The magnitude is modest to allow necessary turns when required, but still biases the policy toward efficient, goal-directed motion.

### 3.5 Learning objective, gradients, and relation to policy-gradient theory

Let $\pi_{\phi }\left(a_{t}|s_{t}\right)$ denote the marginal policy induced by the variational posterior over the policy network weights as in Equation 11.

$$\pi_{\phi }\left(a_{t}|s_{t}\right)=\int p\left.\left(a_{t}|s_{t},W\right)\right)q_{\phi }\left(W\right)dW,$$ (11)

where $q_{\phi }\left(W\right)=N_{v}\left(\mu ,\Sigma \right)$ . With the log-derivative trick, the policy-gradient theorem writes the loss gradient as in Equation 12.

$$\nabla_{\phi }J=E_{\pi_{\phi }}\left[A_{t}\nabla_{\phi }\log\pi_{\phi }\left(a_{t}|s_{t}\right)\right]$$ (12)

where $A_{t}$ is any unbiased advantage estimate. In our implementation $A_{t}$ is the TD error $\delta_{t}=r_{t}\left(s_{t},a_{t}\right)+\gamma V\left(s_{t+1};U\right)-V\left(s_{t};U\right)$ , which is a standard, low-variance advantage estimator.

Because the log function is concave, Jensen gives a lower bound in Equation 13.

$$\log\pi_{\phi }\left(a_{t}|s_{t}\right)\geq E_{N_{v}}\left(\log p\left(a_{t}|s_{t},W\right)\right)\;\text{ELBO on }\log\pi_{\phi }$$ (13)

Maximizing $E_{N_{v}}\left(\log p\left(a_{t}|s_{t},W\right)\right)$ therefore maximizes a *surrogate* for $\log\pi_{\phi }\left(a_{t}|s_{t}\right)$ . Replacing $\log\pi_{\phi }$ with its ELBO inside the actor objective yields the standard advantage-weighted maximum-likelihood surrogate as in Equation 14.

$$L_{\text{Actor}}\left(\phi \right)=\underset{t}{\sum }\delta_{t}\underset{\text{ELBO }-\text{ likelihood}}{\underbrace{E_{N_{v}}\left(\log p\left(a_{t}|s_{t},W\right)\right)}}-\beta \underset{\text{Bayesian Regularizer}}{\underbrace{\text{KL}\left[N_{v}\|N_{p}\right]}},$$ (14)

where β>0 is a regularization weight. This is directly analogous to REINFORCE/actor-critic with (i) an advantage weight $\delta_{t}$ and (ii) a Bayesian regularizer (akin to entropy/trust-region regularization).

The critic or value neural network is trained with the standard TD mean-squared error (MSE) as in Equation 15.

$$L_{\text{Critic}}\left(U\right)=\frac{1}{2}{\left(r_{t}\left(s_{t},a_{t}\right)+\gamma V\left(s_{t+1};U\right)-V\left(s_{t};U\right)\right)}^{2}=\frac{1}{2}\delta_{t}^{2},$$ (15)

and is optimized independently of the actor’s KL/ELBO terms (no gradients from the actor loss flow into U . We use the critic only to supply the advantage estimate $A_{t}=\delta_{t}$ for the actor update; as noted above, $\delta_{t}$ is detached when forming $\nabla_{\phi }L_{\text{Actor}}$ to avoid biasing the critic.

Summary of the learning rule: If we denote the likelihood by the following definition,

$$\ell_{t}\left(\phi ,s_{t}\right)=E_{N_{v}}\left(\log p\left(a_{t}|s_{t},W\right)\right)\approx \log N\left(a_{t};\mu_{a_{t}}\left(s_{t},\phi \right),\Sigma_{a_{t}}\left(s_{t},\phi \right)\right),$$ (16)

where $\mu_{a_{t}},\Sigma_{a_{t}}$ are obtained analytically via our moment-propagation (linear layers and first-order treatment of nonlinearities). The actor gradient is defined in Equation 17.

$$\nabla_{\phi }L_{\text{Actor}}\left(\phi \right)=\underset{t}{\sum }\delta_{t}\nabla_{\phi }\ell_{t}\left(\phi \right)-\beta \nabla_{\phi }\text{KL}\left[N_{v}\|N_{p}\right].$$ (17)

The critic gradient is defined in Equation 18.

$$\nabla_{U}L_{\text{Critic}}\left(U\right)=\delta_{t}\nabla_{U}\left(\delta_{t}\right)\;\text{ with }\delta_{t}\text{ detached in actor updates}.$$ (18)

The interaction of the policy and value networks with the environment and the optimization through the TD error to maximize the cumulative reward is detailed in Algorithm 1.

## 4 Experiments

Algorithm 1 Trust-Nav: Advantage-weighted variational actor–critic with closed-form moment propagation.

1: **Inputs:** Number of episodes, K , maximum number of steps per episode T , learning rate η , discount factor γ , KL weight β , and the initial conditions for the learnable parameters ϕ={μ,Σ} , and U .
2: **Init**: Variational policy: $q_{\phi }\left(W\right)=N_{v}\left(\mu ,\Sigma \right)$ , and the value (critic) network parameters U .
3: **for** k=1 to K **do**
4:     Reset the environment to get the initial state $s_{0}$

5:    **for** t=1 to T **do**
6:       **Policy forward**: propagate posterior means/covariances $N_{v}\left(\mu ,\Sigma \right)$ through the policy network
         to get $p\left(a_{t}|s_{t},W\right)$ .
7:        **Action selection**: sample $a_{t}∼N\left(\mu_{a_{t}},\Sigma_{a_{t}}\right)$

8:        **Execute** $a_{t}$ and observe the reward $r_{t}\left(s_{t},a_{t}\right)$ and the new state $s_{t+1}$

9:        **Value forward**: forward pass through the value network, and calculate $V\left(s_{t};U\right)$ and critic TD
           error $\delta_{t}=r_{t}\left(s_{t},a_{t}\right)+\gamma V\left(s_{t+1};U\right)-V\left(s_{t};U\right)$

10:        **Critic loss**: $L_{\text{Critic}}\left(U\right)=\frac{1}{2}\delta_{t}^{2}$ .
11:        **Actor loss**: $L_{\text{Actor}}\left(\phi \right)=$ $\delta_{t}E_{N_{v}}\left(\log p\left(a_{t}|s_{t},W\right)\right)-\beta \text{KL}\left[N_{v}\|N_{p}\right]$ .
12:        **Update the value (critic) network parameters**: $U\leftarrow U-\eta \nabla_{U}\;L_{\text{Critic}}\left(U\right)$ .
13:        **Update the variational policy network parameters**: $\phi \leftarrow \phi -\eta \nabla_{\phi }\;L_{\text{Actor}}\left(\phi \right)$ .
14:    **end for**
15: **end for**

### 4.1 Experimental set-up

In our experiments, we utilize OpenAI’s Gym-Gazebo extension (Zamora et al., 2016), which leverages the Gazebo robotics simulator to provide a standardized and reproducible interface for reinforcement learning in robotic environments. The Gym-Gazebo extension library facilitates the creation of simulated environments where robotic agents are readily accessible and can be seamlessly integrated with machine learning architectures for both training and evaluation (Zamora et al., 2016). This simulator enables precise control of environmental conditions and sensor characteristics, which is essential for isolating and quantifying the effects of uncertainty modeling in our framework.

The proposed Trust-Nav model is deployed and evaluated using a simulated *TurtleBot3* robot and pre-configured environments provided by OpenAI’s repositories. The action space consists of three discrete actions: move forward, turn left, and turn right, with fixed linear and angular velocities defined in the TurtleBot3 simulation. The state representation comprises processed 2D LiDAR scan data (360° range readings) and robot pose estimates from ROS, all normalized to [0,1] for stable learning.

Experimental results are systematically documented and analyzed in comparison to a carefully selected baseline model—Det-Nav—which represents a deterministic navigation approach. Both Trust-Nav and Det-Nav share the same underlying network architecture; however, Det-Nav does not incorporate variational inference and instead relies on point estimates for action selection, omitting the propagation of uncertainty through the policy network. This comparison allows us to isolate and assess the impact of uncertainty modeling on decision-making, particularly in the presence of environmental noise or corruption, thereby validating the robustness and effectiveness of the proposed Trust-Nav approach. This controlled architectural parity allows us to isolate the contribution of uncertainty modeling to policy performance, avoiding confounding effects from differences in mapping, planning, or control modules.

Both Trust-Nav and Det-Nav models employ identical 10-layer convolutional neural network (CNN) architectures for both the policy and value networks. The architecture begins with three convolutional layers using 32 filters of size 5×5 , followed by three layers with 64 filters of size 3×3 . This is succeeded by three additional convolutional layers with 128 filters of size 1×1 , which capture fine-grained spatial features. The final layer is a fully connected layer that produces the output corresponding to either the policy distribution or the value estimate, depending on the network’s role. While both Trust-Nav and Det-Nav share identical network architectures and the same hyperparameter search protocol, the final learning rates differ due to independent tuning for stable convergence in each method. This approach avoids biasing the comparison by forcing identical learning rates despite differing optimization dynamics (variational inference in Trust-Nav vs. point estimates in Det-Nav). All hyperparameter values are provided in Table 1 for full transparency.

**TABLE 1:** Hyperparameters for the Trust-Nav and Det-Nav models.

Hyperparameter	Trust-Nav	Det-Nav
Learning rate (η)	0.0002	0.001
Batch size	16	16
Discount factor (γ)	0.95	0.95
Replay memory	100,000	100,000
Episode size	1,500 steps	1,500 steps
Total number of episodes	200	200
Exploration decay rate	0.999	0.999

Noise and disturbance analysis is conducted using realistically parameterized Gaussian noise and adversarial attacks (Table 2), with values chosen to reflect ranges reported for common mobile robot sensors such as LiDAR and RGB-D cameras.

**TABLE 2:** Noise levels for random (Gaussian) noise and adversarial attacks.

Type of noise	Levels of noise						
Random noise (std)	0.0001	0.001	0.1	0.2	0.3	0.4	0.5
Adversarial noise (ε)	0.0001	0.001	0.01	0.05	0.1	–	–

**FIGURE 3:**
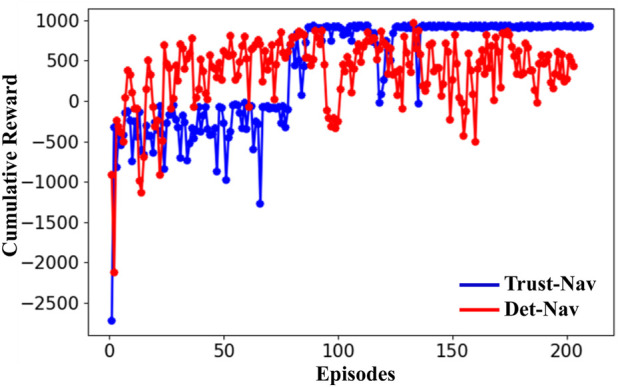
The cumulative reward of the proposed Trust-Nav (blue curve) and Det-Nav (red curve) in a noise-free test environment (without adding noise).

**FIGURE 4:**
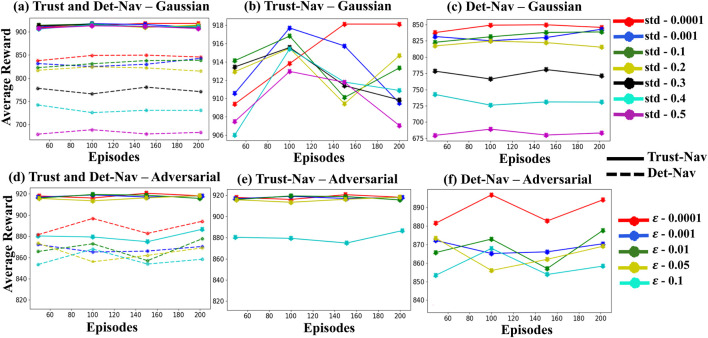
The average reward values of Trust-Nav compared to Det-Nav, validated on Gazebo environments under various levels of Gaussian noise and adversarial attacks. **(a)** Both models are evaluated under Gaussian noise. **(b)** Trust-Nav is tested under Gaussian noise. **(c)** Det-Nav is tested under Gaussian noise. **(d)** Both models are evaluated under adversarial attacks. **(e)** Trust-Nav is tested under adversarial attacks. **(f)** Det-Nav is tested under adversarial attacks.

The experimental pipeline is implemented using the Robot Operating System (ROS), which runs on a Linux-based system with computation accelerated by four NVIDIA Quadro RTX 6000 GPUs (24 GB memory each). Each policy is evaluated over 200 independent episodes per condition to ensure statistical reliability and consistency of results. To assess learning stability and navigation robustness, we track key performance metrics, including *Moving Average Rewards, Maximum Rewards, and Cumulative Rewards*. These metrics are summarized in Figures 3, 4 and Tables 3, 4.

**TABLE 3:** Maximum and average reward values for the Trust-Nav and Det-Nav models in test environments corrupted with different levels of Gaussian noise (std).

Noise level (std)	Trust-Nav		Det-Nav	
	Max reward	Avg reward	Max reward	Avg reward
0.0001	916.700 ± 1.31	916.316 ± 1.23	846.197 ± 4.56	836.894 ± 4.98
0.001	915.423 ± 2.65	915.313 ± 1.92	838.760 ± 4.89	832.734 ± 5.87
0.1	914.288 ± 2.28	913.596 ± 2.21	832.301 ± 5.69	825.627 ± 5.23
0.2	913.864 ± 2.91	913.379 ± 2.29	808.853 ± 6.98	812.423 ± 6.94
0.3	912.926 ± 3.17	913.127 ± 2.54	771.164 ± 8.79	774.136 ± 7.49
0.4	909.846 ± 2.94	912.864 ± 3.62	730.738 ± 8.96	734.537 ± 8.89
0.5	909.011 ± 3.52	911.365 ± 3.48	683.200 ± 9.25	682.930 ± 10.61

**TABLE 4:** Maximum and average reward values for the Trust-Nav and Det-Nav models in test environments corrupted with different levels of adversarial attacks (ε) .

Noise level (ε)	Trust-Nav		Det-Nav	
	Max reward	Avg reward	Max reward	Avg reward
0.0001	921.269 ± 2.12	918.213 ± 1.97	883.674 ± 5.89	878.989 ± 5.51
0.001	918.518 ± 2.11	917.156 ± 2.36	877.642 ± 5.88	868.454 ± 6.54
0.01	917.844 ± 1.98	916.469 ± 2.58	873.430 ± 6.97	863.324 ± 6.94
0.05	915.770 ± 2.56	913.695 ± 2.49	868.769 ± 6.82	862.221 ± 6.99
0.1	886.264 ± 2.96	880.293 ± 3.12	853.136 ± 9.83	818.373 ± 8.85

### 4.2 Robustness analysis under noisy conditions

We evaluate the robustness of the proposed Trust-Nav model against two well-defined disturbance types: additive Gaussian noise and adversarial attacks, comparing it to the Det-Nav model. The post-training robustness analysis is performed after the models are fully trained and validated in a simulated training environment, which ensures that the performance degradation can be attributed purely to test-time perturbations, without affecting the learned policy during training. We design the experiments such that we start training the robot in a clean, noise-free environment before introducing noise to assess the effect of noise on policy performance without influencing the learning process. Then, we incrementally introduce noise complexity in a test environment using random (Gaussian) noise and adversarial attacks to progressively degrade the robot’s perception. First, we evaluate the performance of the proposed Trust-Nav compared to Det-Nav models in a clean test environment (without noise). Then, we gradually add various levels of Gaussian or adversarial noise to the test environment states to evaluate the performance of each model.

**FIGURE 5:**
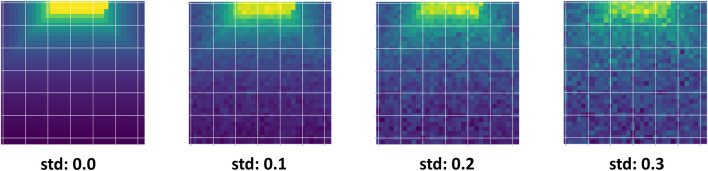
Depth camera observations under varying input noise levels used for robot navigation. The standard deviation (std) values (0.0, 0.1, 0.2, 0.3) represent increasing amounts of Gaussian perturbation added to the depth measurements. As noise grows, the sensor data becomes progressively more corrupted, highlighting the challenge of robust policy learning under uncertain perception.

Gaussian noise is introduced in seven levels of increasing severity, defined by the standard deviation (std) parameter, which is chosen to align with empirical sensor noise characteristics documented in robotics literature. Figure 5 demonstrates the depth camera observations for robot navigation under increasing Gaussian noise levels, where higher standard deviations progressively degrade the visual quality of the input. As shown in the figure, higher noise levels progressively corrupt the sensor observations, making navigation more challenging. This experiment demonstrates how Trust-Nav adapts to noisy depth measurements by leveraging uncertainty propagation in its policy.

Adversarial examples are generated using the Fast Gradient Sign Method (FGSM) (Goodfellow et al., 2015), with five attack levels controlled by ε as in Equation 19. Both disturbance types are applied in the test environment only, preserving a clean training phase for fair assessment.

$$s_{t}^{\text{adv}}=s_{t}+\varepsilon \cdot \text{sign}\left(\nabla_{s_{t}}\ell_{t}\left(\phi ,s_{t}\right)\right),\;\text{where }\|s_{t}^{\text{adv}}\|\in \left[0,1\right].$$ (19)

Here, $\ell_{t}$ is the ELBO likelihood of the policy network defined in Equation 16, while the networks’ parameters will be frozen during attack generation. The normalization constraint ensures that adversarial states remain in the valid input range [0,1] , i.e., $\|s_{t}+\varepsilon \text{ sign}\left[\nabla_{s_{t}}\;\ell_{t}\left(\phi ,s_{t}\right)\right]\|\in \left[0,1\right]$ . Table 2 provides ε values for the five levels of attacks applied to the test environment. Both Trust-Nav and Det-Nav models are validated under all noise levels, with each level undergoing 200 episodes per run, with results averaged to ensure consistency. The complete noise/attack specifications are given in Table 2.

### 4.3 Robot uncertainty vs. signal to noise ratio

The proposed Trust-Nav framework develops a robot that produces actions and uncertainty information simultaneously in the form of the actions’ distribution mean and variance-covariance matrix. The analysis of uncertainty under noisy conditions (when the environment is corrupted by Gaussian noise or adversarial attacks) provides insights into the navigation performance after deployment and possible detection of the robot’s failure due to environment complexity. We analyze the predictive variance of actions at various levels of Gaussian noise and adversarial attacks. The amount of noise at each level is measured using the signal-to-noise ratio (SNR). For adversarial attacks, the signal is the clean input state $s_{t}$ , and the noise is the perturbation vector $\varepsilon \cdot \text{sign}\left(\nabla_{s_{t}}\ell_{t}\left(\phi ,s_{t}\right)\right)$ applied by FGSM. The SNR is typically defined in decibels (dB) as in Equation 20.

$$\text{SNR}\left(\varepsilon \right)=10\log_{10}\left(\frac{\|s_{t}{\|}_{2}^{2}}{\|\varepsilon \cdot \text{sign}\left(\nabla_{s_{t}}\ell_{t}\left(\phi ,s_{t}\right)\right){\|}_{2}^{2}}\right).$$ (20)

**FIGURE 6:**
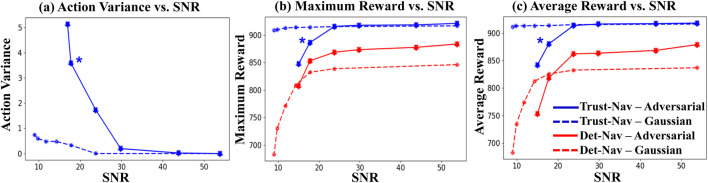
Relationship between signal-to-noise ratio (SNR) and **(a)** average action variance, **(b)** maximum episode reward, and **(c)** average episode reward for Trust-Nav and Det-Nav under Gaussian noise and adversarial perturbations. Blue curves correspond to Trust-Nav and red curves to Det-Nav; solid lines indicate adversarial attacks, and dashed lines indicate Gaussian noise. Higher action variance at low SNR reflects increased navigation uncertainty, with Trust-Nav showing a statistically significant increase in variance compared to noise-free conditions (Wilcoxon signed-rank test, p<0.01 ). Trust-Nav consistently maintains higher maximum and average rewards across all noise conditions, demonstrating robustness to both Gaussian and adversarial perturbations as compared to the Det-Nav baseline. The star marker denotes the point of statistically significant variance increase.

The average action variance is calculated for all the test frames at each noise level. We scale the action variance curves from zero by subtracting the variance at the baseline (clean test environment states without noise) at each level. The resulting average action variance is plotted against the respective SNR values to produce *variance-vs-SNR* curves (Figure 6), which are interpreted from right to left. The variance values at the extreme right side of the graph correspond to very high SNR (low noise levels). The addition of noise results in a decrease in the SNR values, progressing from right to left. The extreme left point represents the average variance at the lowest SNR (i.e., the highest levels of noise).

## 5 Results and discussion

### 5.1 Performance analysis and robustness

This section discusses the performance evaluation and the robustness behavior of the proposed Trust-Nav model compared to the baseline Det-Nav model. The average, cumulative, and maximum rewards demonstrate the performance metric of the models in the test-simulated environment. Figure 3 illustrates the cumulative reward obtained by the proposed Trust-Nav (blue curve) and Det-Nav (red curve) in a noise-free test environment (without adding noise). Initially, both models yield low reward values; however, as training progresses over multiple episodes, the rewards steadily increase, indicating effective policy learning and successful maximization of the reward function.

Figure 4 presents the average reward values for Trust-Nav and Det-Nav models in a test simulated environment under varying levels of Gaussian noise and adversarial attacks. Each curve represents the average reward obtained in a separate experiment corresponding to a specific noise level. Figures 4b,c show the average rewards of Trust-Nav and Det-Nav, respectively, across Gaussian noise levels ranging from 0.0001 to 0.5. In Figure 4a, the average rewards of both models are plotted together for direct comparison, with solid lines representing Trust-Nav and dashed lines representing Det-Nav. As expected, increasing the standard deviation of the injected Gaussian noise has a negative impact on performance for both models. However, Trust-Nav demonstrates greater robustness by consistently achieving higher average rewards—approximately 900—compared to Det-Nav, whose performance drops to around 675 under high noise conditions.

Figures 4e,f demonstrate the rewards achieved by Trust-Nav and Det-Nav models, respectively, when adversarial perturbations are introduced into the environment’s state observations at varying levels of attack severity ( ε=0.0001 - ε=0.1 ). Every curve presents an experiment with distinct attack severity. Figure 4d provides a comparative view, plotting the reward trajectories of both models under all five levels of adversarial attacks. In this figure, solid lines represent Trust-Nav, while dashed lines represent Det-Nav. To ensure visual consistency and facilitate comparative analysis, the same color scheme is used across all subplots to indicate equivalent noise or attack severity levels.

As expected, the introduction of adversarial examples negatively impacts both models, with increasing attack strength leading to greater reward degradation. Nevertheless, Trust-Nav exhibits significantly more robust behavior under adversarial conditions. Its average reward remains relatively stable across all but the highest attack level, decreasing only slightly from approximately 920 to 880 when (ε=0.1) . In contrast, Det-Nav exhibits a more pronounced decline, with reward values decreasing from approximately 900 to 850 under the same conditions. These results highlight the enhanced robustness and reliability of Trust-Nav to adversarial perturbations in comparison to its deterministic counterpart.

### 5.2 Robot uncertainty analysis and self-assessment

We employ the action variance at the output of the variational policy network in the Trust-Nav model as a quantitative metric to evaluate the robot’s navigation confidence (or uncertainty) without requiring any additional sensing, data processing or computational overhead. This property enables what we refer to as *self-assessment*, whereby the model internally gauges the trustworthiness of its own actions based on the magnitude of the output variance. Intuitively, higher action variance reflects increased uncertainty in navigation decisions, signaling low confidence in the robot’s actions under challenging or degraded sensing conditions.

Figure 6 illustrates the relationship between signal-to-noise ratio (SNR) and (a) average action variance, (b) maximum episode reward, and (c) average episode reward for both Trust-Nav and the deterministic baseline Det-Nav. Blue curves represent Trust-Nav and red curves represent Det-Nav, with solid lines denoting adversarial perturbations and dashed lines denoting Gaussian noise. The plots read from right to left, as lower SNR values correspond to higher noise levels.

Across all noise levels, Trust-Nav consistently outperforms Det-Nav in both maximum and average rewards. While both models experience declining performance at low SNR, Trust-Nav maintains significantly higher rewards, particularly under Gaussian noise, where the average reward decreases by only ( ≈ 0.5%) compared to ( ≈ 14%) for Det-Nav. Under adversarial perturbations, Trust-Nav experiences a larger drop ( ≈ 8%) but still remains superior to Det-Nav’s ( ≈ 18%) reduction. Importantly, under low SNR (e.g., SNR <20 dB), the action variance of Trust-Nav increases sharply, indicating heightened uncertainty that correlates with performance degradation. This relationship is statistically significant according to a Wilcoxon signed-rank test (p<0.01) when comparing action variance at high versus low SNR, validating variance as a meaningful uncertainty indicator. We refer to the point of statistically significant variance increase by a star in Figure 6.

The increase in variance concurrent with declining reward demonstrates that Trust-Nav is self-aware of deteriorating navigation performance. This self-assessment capability is a key step toward safe and reliable deployment in real-world robotics, where the ability to detect and respond to uncertain decision states is essential for preventing unsafe actions.

### 5.3 Discussion

This paper introduces a new deep reinforcement learning navigation (Trust-Nav) framework that propagates variational moments through the policy neural network and estimates the uncertainty in the robot’s actions and localization. The variational policy network propagates the first two moments (mean and covariance) of the variational posterior distribution of the network’s parameters and estimates the uncertainty in the robot’s actions via the variance of the policy distribution. We conduct a comprehensive analysis using the Gazebo simulated environment under various noisy conditions. The performance of the Trust-Nav model is compared with the state-of-the-art DRL navigation networks under multiple levels of Gaussian noise and adversarial attacks, i.e., FGSM.

Our analysis reveals that the Trust-Nav model maintains its reward values and outperforms the corresponding deterministic DRL navigation when the environment is subject to Gaussian noise or adversarial attacks. Furthermore, the robot’s action variance significantly increases when the adversarial noise is high, and the model’s reward values start to decrease. The moments of the policy variational distribution transmit vital state features from the environment through the policy network to the action predictions. The second moment (i.e., the variance) of the variational distribution over the policy parameters filters the state features according to their importance. This policy filtering mechanism of the environmental dynamic features via the variance of the variational distribution forces the robot’s action variance to increase when these features are corrupted with noise or adversarial attacks.

In addition to the quantitative results, we also observe qualitative behavioral patterns that reinforce the role of action variance as a self-assessment signal. For instance, under high-uncertainty zones corresponding to low-SNR adversarial conditions, the robot exhibits noticeably cautious navigation—slowing down, hesitating before turns, and occasionally failing to commit to decisive maneuvers. These behaviors coincide with spikes in action variance, highlighting the model’s internal recognition of unreliable decision states. Conversely, when operating in higher-SNR conditions, the variance remains low, and the robot navigates confidently, with smoother trajectories and fewer hesitations. This qualitative evidence illustrates how Trust-Nav’s uncertainty-aware design enables the robot to adaptively signal and respond to reliability degradation, offering an interpretable connection between statistical variance and observable robot performance.

### 5.4 Deployment perspective and real-world applicability

Although our evaluation is conducted in simulation, the Trust-Nav framework is designed with deployment feasibility in mind. By explicitly propagating both the mean and variance of the variational posterior through the policy network, the approach enables the robot to self-assess the reliability of its actions in real time, without introducing additional computational burden or requiring external supervision. This self-assessment capability is particularly advantageous for physical deployment, as it allows the robot to identify low-confidence states and adapt its behavior accordingly, thus enhancing safety in uncertain or adversarial environments. Importantly, because the proposed method operates directly on the learned policy outputs, it is agnostic to the underlying robot platform and sensing configuration, which facilitates seamless transfer from simulation to hardware. This positions Trust-Nav as a practical framework for bridging robust uncertainty-aware navigation with real-world autonomous systems.

## 6 Conclusion

We propose Trust-Nav, a deep reinforcement learning framework that incorporates uncertainty estimation via a variational policy network. The proposed Trust-Nav is built on fundamental principles of Bayesian density propagation in dynamical systems. By propagating moments of the variational policy network, Trust-Nav enables robust decision-making and provides a built-in measure of action confidence (or equivalently uncertainty). Experiments in simulated environments demonstrate that Trust-Nav model consistently outperforms baseline models and remains robust under Gaussian noise and adversarial attacks. Trust-Nav models maintain not only higher rewards but also demonstrate reduced sensitivity to input corruption. When the reward values decrease due to the high level of adversarial attacks, the uncertainty associated with the robot’s actions increases significantly to warn the robot of uncertain actions. This integration of uncertainty into the policy network promotes safer and more reliable navigation, especially in complex or unpredictable environments. Trust-Nav offers a step toward deployable, self-aware robotic systems capable of recognizing and responding to their own limitations.

## 7 Future work

While the present study introduces closed-form variational moment propagation within DRL policy networks—offering a tractable and sampling-free approach to uncertainty estimation—several extensions are envisioned to further enhance the framework’s accuracy and applicability. First, the current formulation adopts an independence assumption for network parameters across and within layers to ensure scalability and real-time feasibility. In future work, we plan to investigate structured covariance approximations, such as Kronecker-factored or low-rank representations, to capture inter-parameter correlations while preserving computational efficiency. Second, our method currently employs a first-order Taylor approximation for nonlinear activation functions. Although this enables a closed-form, low-latency uncertainty propagation, we will explore the use of unscented transformations, which can approximate nonlinear mappings up to second-order accuracy, thereby reducing approximation error without resorting to Monte Carlo sampling. Finally, future studies will expand the evaluation to include real-world robotic platforms, additional noise models derived from real sensor data, and comparisons with other uncertainty-aware DRL approaches, further validating the robustness and generalizability of the proposed framework.

## Data Availability

The datasets analyzed for this study are available in the gym-gazebo repository at https://github.com/erlerobot/gym-gazebo All source code, experiment configurations, and instructions to reproduce the results are publicly available at: https://github.com/dimahdera/Robust-Uncertainty-Estimation-Framework-in-Deep-Reinforcement-Learning-for-Active-SLAM.git
